# A complex intervention to improve implementation of World Health Organization guidelines for diagnosis of severe illness in low-income settings: a quasi-experimental study from Uganda

**DOI:** 10.1186/s13012-017-0654-0

**Published:** 2017-11-06

**Authors:** Matthew J. Cummings, Elijah Goldberg, Savio Mwaka, Olive Kabajaasi, Eric Vittinghoff, Adithya Cattamanchi, Achilles Katamba, Nathan Kenya-Mugisha, Shevin T. Jacob, J. Lucian Davis

**Affiliations:** 1 Division of Pulmonary, Allergy, and Critical Care Medicine, Columbia University Medical Center, New York, NY USA; 2 ImpactMatters, New York, NY USA; 3 Walimu, Kampala, Uganda; 4 Department of Epidemiology and Biostatistics, University of California San Francisco School of Medicine, San Francisco, CA USA; 5 Division of Pulmonary and Critical Care Medicine, University of California San Francisco School of Medicine, San Francisco, CA USA; 6 School of Medicine, Makerere University College of Health Sciences, Kampala, Uganda; 7 Division of Allergy and Infectious Diseases, University of Washington School of Medicine, Seattle, WA USA; 8 Department of Epidemiology of Microbial Diseases, Yale University School of Public Health, New Haven, CT USA; 9 Section of Pulmonary, Critical Care, and Sleep Medicine, Yale University School of Medicine, New Haven, CT USA

**Keywords:** Implementation, Quality improvement, Critical care, Africa South of the Sahara, Uganda, Global health

## Abstract

**Background:**

To improve management of severely ill hospitalized patients in low-income settings, the World Health Organization (WHO) established a triage tool called “Quick Check” to provide clinicians with a rapid, standardized approach to identify patients with severe illness based on recognition of abnormal vital signs. Despite the availability of these guidelines, recognition of severe illness remains challenged in low-income settings, largely as a result of infrequent vital sign monitoring.

**Methods:**

We conducted a staggered, pre-post quasi-experimental study at four inpatient health facilities in western Uganda to assess the impact of a multi-modal intervention for improving quality of care following formal training on WHO “Quick Check” guidelines for diagnosis of severe illness in low-income settings. Intervention components were developed using the COM-B (“capability,” “opportunity,” and “motivation” determine “behavior”) model and included clinical mentoring by an expert in severe illness care, collaborative improvement meetings with external support supervision, and continuous audits of clinical performance with structured feedback.

**Results:**

There were 5759 patients hospitalized from August 2014 to May 2015: 1633 were admitted before and 4126 during the intervention period. Designed to occur twice monthly, collaborative improvement meetings occurred every 2–4 weeks at each site. Clinical mentoring sessions, designed to occur monthly, occurred every 4–6 months at each site. Audit and feedback reports were implemented weekly as designed. During the intervention period, there were significant increases in the site-adjusted likelihood of initial assessment of temperature, heart rate, blood pressure, respiratory rate, mental status, and pulse oximetry. Patients admitted during the intervention period were significantly more likely to be diagnosed with sepsis (4.3 vs. 0.4%, risk ratio 10.1, 95% CI 3.0–31.0, *p* < 0.001) and severe respiratory distress (3.9 vs. 0.9%, risk ratio 4.5, 95% CI 1.8–10.9, *p* = 0.001).

**Conclusions:**

Theory-informed quality improvement programs can improve vital sign collection and diagnosis of severe illness in low-income settings. Further implementation, evaluation, and scale-up of such interventions are needed to enhance hospital-based triage and severe illness management in these settings.

**Trial registration:**

Severe illness management system (SIMS) intervention development, ISRCTN46976783

**Electronic supplementary material:**

The online version of this article (10.1186/s13012-017-0654-0) contains supplementary material, which is available to authorized users.

## Background

Globally, the burden of severe illness is concentrated in low-income countries where the prevalence of sepsis, shock, and severe respiratory infections is high and associated mortality is substantial [1, 2]. In contrast to high-income settings where severely ill patients are cared for in dedicated intensive care units, management of severe illness in low-income settings is often carried out on general hospital wards under the same resource constraints that exist for other hospitalized patients [3, 4].

To improve management of severely ill hospitalized patients in low-income settings, the World Health Organization (WHO), through its program on the Integrated Management of Adolescent and Adult Illness (IMAI), developed the District Clinician Manual and a triage tool called “Quick Check” to provide clinicians with a rapid, standardized approach to identifying patients with severe illness based on recognition of abnormal vital signs [5]. A training course called “Quick Check+” has been developed to facilitate instruction by the use of the tool.

Although clinician training is an integral component of guideline implementation, available data suggest that multi-modal strategies guided by local experience and a validated theory of practice change are needed to facilitate sustained implementation of standardized interventions [6, 7]. A cluster-randomized evaluation of the implementation of the related Integrated Management of Childhood Illness program demonstrated that combining clinician training with local facilitation, external supervision, and face-to-face feedback resulted in sustained improvements in the management of severely ill children compared to an implementation strategy involving only passive dissemination of guidelines and minimal face-to-face engagement with trainees [8].

In an effort to strengthen the impact of the IMAI Quick Check + program on the management of severely ill patients in Uganda, we developed the Severe Illness Management System (SIMS) platform, a multi-modal quality improvement program using a theory-informed approach to implementation. Here, we report the development, implementation, and impact of the SIMS intervention on vital sign collection and diagnosis of severe illness conditions among patients hospitalized at four inpatient health facilities in western Uganda.

## Methods

### Study sites and participants

We introduced the SIMS platform during roll-out of the Quick Check + training program at eight health facilities in western Uganda chosen by the Uganda Ministry of Health. From the pool of health facilities that participated in training, we selected three general hospitals and one inpatient community health center as study sites (Additional file 1: Table S1). We chose these health facilities based on the availability of logistical support for training and the presence of an existing hospital quality improvement team. The population of the district where these health facilities are located is approximately 695,000, and HIV prevalence is 4% [9].

We reviewed medical charts of consecutive adult and adolescent (age ≥ 14 years) patients admitted to the general medical wards through the casualty department at each health facility. We excluded patients whose primary admitting diagnosis was an emergent surgical or obstetrical condition.

### Quick Check + training program

The Quick Check + training program features clinical instruction in early recognition and emergent management of four severe illness conditions: undifferentiated shock, sepsis, severe respiratory distress, and altered consciousness (Additional file 1: Table S2). The course targets staff at general hospitals in resource-limited settings (e.g., hospitals that do not routinely provide mechanical ventilation [except during surgery] or invasive hemodynamic monitoring). The Quick Check + training lasted 5 days with approximately 3 days covering recognition and immediate management of emergency vital signs and 2 days covering management of specific severe illness conditions. The training was implemented in collaboration with the local facilitators trained by the IMAI Alliance, an international non-governmental organization.

### Severe Illness Management System (SIMS) intervention development

To strengthen the impact of the IMAI Quick Check + program, we developed the SIMS platform, our multi-modal approach to program implementation, using the Behavior Change Wheel framework and the COM-B (“capability,” “opportunity,” and “motivation” determine “behavior”) model. The Behavior Change Wheel and COM-B model is an implementation framework chosen for a number of features that maximize its usability in routine public health practice. First, it was developed through a systematic review of 19 existing implementation frameworks to produce a comprehensive list of intervention descriptors at a level of generality that is usable by intervention designers and policy makers [10, 11]. Second, it incorporates a comprehensive, coherent, and overarching model of behavior change, providing a basis for targeting interventions to underlying barriers [10, 11]. Third, in previous work in Uganda, we have found that it is a framework that can be readily understood and adopted by clinicians and public health practitioners without a background in health psychology or implementation science [12]. Practically, our prior work caring for severely ill patients in resource-limited settings, coupled with the available literature, informed our initial understanding of barriers to optimal management of severely ill patients in Uganda [13–15]. In this context, the COM-B model identifies clinician motivation (e.g., wants, needs, beliefs, plans) as the primary driver of practice change, but motivation is dependent on clinicians having the capability (e.g., memory, knowledge, and skills) and opportunity *(*e.g., time, space, equipment, supplies) to facilitate change (Additional file 1: Figure S1) [10, 11, 16].

To identify sources of particular behaviors that might serve as productive targets for intervention, program officers evaluated implementation barriers in each of the three domains of the COM-B system. The capacity of health workers to carry out guideline-based practices was assessed in pre- and post-training surveys during the Quick Check + training. To assess opportunity barriers, they surveyed physical resources using a standardized tool and carried out a systematic environmental assessment of the patient flow practices through activity mapping exercises and direct observation. To assess motivation barriers, focus group discussions were carried out with clinicians using standardized guidelines designed to assess baseline patterns of practice, followed by solicitation of ideas on how to transform the pattern of practice to meet Quick Check + standards.

Subsequently, we selected the modalities in our intervention based on their intrinsic functionality, as conceptualized in the Behavior Change Wheel. We then organized them according to the following domains of the COM-B model and intervention functions of the Behavior Change Wheel: (a) providing structured training in severe illness care and making clinical practice guidelines available to clinicians via electronic tablets, in order to improve the *capability* of clinicians to care for severely ill patients; (b) reorganizing care processes and staff responsibilities to give clinicians more *opportunity* to provide high-quality care to severely ill patients, by ensuring availability of all basic equipment and supplies needed to deliver severe illness care, by increasing the time available for each core component activity, and by increasing the efficiency with which these activities are carried out; and (c) enhancing *motivation* to provide high-quality severe illness care by empowering local clinicians and by providing routine performance feedback and clinical mentoring.

### SIMS intervention design

SIMS comprises both a training phase and a post-training reinforcement phase. Prior to the introduction of SIMS, SIMS program managers with training in social sciences and hospital administration (O.K., N.K.M., S.M.) worked with facility staff at each site to identify barriers to diagnosing and managing severe illness. This assessment included systematically documenting observations about the physical setting and equipment at each site and reviewing existing clinical processes for delivering severe illness care (Additional file 1: Appendix S1 and Appendix S2). Specific barriers identified across facilities included inadequate skills reported by hospital staff in recognition and resuscitation of severely ill patients, absence of designated areas for emergency care, limited staffing in existing emergency care areas (i.e., outpatient triage and casualty departments), and limited equipment for vital sign collection (i.e., thermometers, blood pressure cuffs, pulse oximeters, watches, and wall clocks to record time) (Additional file 1: Table S3a). During the training phase, staff at each facility developed a site-specific quality improvement plan to address the identified barriers.

Following the training phase, we implemented three key behavior change interventions derived from the Behavior Change Wheel and COM-B model, using a Plan-Do-Study-Act (PDSA) learning cycle to execute the quality improvement plan [17]. These interventions included collaborative improvement meetings, clinical performance audits and feedback, and clinical mentoring (Table 1 and Additional file 1: Table S3b). During collaborative improvement meetings, the SIMS program managers worked with hospital staff led by a locally designated clinician serving as a “champion” tasked with leading efforts to implement the quality improvement plan. These meetings included a brief review of recent data audit reports and the action plan from the previous meeting, followed by the creation of a new action plan to be pursued during the next “Do” cycle. For example, in one health facility, an unused room in the outpatient department previously utilized for injections was re-organized into a triage and resuscitation bay. At another facility, with support of the hospital administration, staff members were re-assigned to cover emergency care areas when duties elsewhere were unoccupied (Additional file 1: Table S3b). Throughout the study period, a discretionary fund of USD 1500 was also provided to each facility to support restructuring of the physical environment to aid plan implementation. The fund was used primarily to purchase vital sign monitoring equipment and repair areas to be used for emergency care (Additional file 1: Table S3b).

**Table 1:** Components of the SIMS intervention, specified according to the Template for Intervention Description and Replication (TIDieR) Checklist

SIMS components	Collaborative improvement meetings	Clinical performance audits and feedback	Clinical mentoring
Why	To guide facility staff on implementing collaborative improvement plans	To assess how the Quick Check is applied in local practice settings and reinforce the need for high performance on specific quality indicators derived from the Quick Check	To guide clinicians on application of Quick Check in local practice settings
What	Systematic assessment of local resources for severe illness management, goal setting by facility stakeholders with external supervision, and group problem-solving	Comprehensive monitoring of clinical performance through daily medical record extraction, with mentored review of regular performance indicator reports	Bedside teaching rounds and mentored reviews of clinical cases; simulation sessions for medical ward teams
Who provides	Local champion	On-site data collectors and project manager	Visiting expert clinician
How	In person with all clinical, administrative, and support staff	Data collection using CommCare, an open-source data collection platform; comprehensive performance reports delivered to clinical leaders at each site via email; focused messages about specific performance indicators delivered to individual clinical staff via SMS	Shadowing at the bedside
Where	At the hospital	At the hospital	At the hospital
When and how much	One hour twice a month	Email reports weekly SMS weekly	One full day each month
Tailoring	We added a USD 1500 process improvement fund to enable facilities to act on improvement priorities.	The program manager developed reports and sent SMS using DHIS2, an open-source electronic health record platform approved by the Ministry of Health.	None
Modifications	The clinical team used the pre-training facility assessment report to develop a work plan for quality improvement.	The clinical leader discussed the reports with staff during collaborative improvement meetings.	None
How well	At least monthly at all 4 sites, bi-monthly at 2 sites	As designed	Every 4–6 months

*Abbreviations*: *DHIS2* District Health Information System Version 2 (Oslo, Norway), *SMS* short messaging service

For audits and feedback, a program manager (S.M.) used an electronic data management platform (see “Data collection”) to generate and send focused weekly updates to clinicians via text messages highlighting specific clinical management indicators. The program manager also sent comprehensive weekly reports on all performance indicators to each site’s clinical leader via email. Site leaders shared and discussed the reports with facility staff at bi-monthly meetings where they also reviewed progress on collaborative improvement work plans, and set goals for future performance.

Finally, to reinforce management principles emphasized during the training phase, expert clinicians visited each hospital to provide clinical mentoring and to conduct simulation sessions for medical ward teams.

### Staggered, pre-post quasi-experimental study design

Prior to initiating our study, we held meetings with the leadership teams at each health facility to explain our objectives in implementing the SIMS interventions and evaluating their ability to improve diagnosis of severe illness. We then invited selected staff from each facility (two clinicians, two nurses, two managers, and two support staff from each site) to attend Quick Check + training prior to initiating data collection at which time physical copies of Quick Check + guidelines were provided. Subsequently, we implemented the SIMS platform using a quasi-experimental, staggered, pre-post design. We chose this design for ethical and logistical reasons, not wanting to withhold the SIMS platform given its potential benefits for improving severe illness care to the level of WHO-recommended standards. However, because we lacked the human capacity to implement SIMS at all sites simultaneously, we randomly assigned the sequence for introducing the SIMS interventions, with a new site launching approximately every 6 weeks and an a priori plan to launch two adjacent sites simultaneously (Additional file 1: Figure S2).

### Data collection

Using structured data collection forms on electronic tablets (CommCare, Dimagi, Cambridge, MA, USA), three trained assistants gathered demographic and clinical data and in-hospital outcome data through daily review of medical charts. Data were uploaded wirelessly to a remote, secure server and transferred into an interoperable electronic data platform approved by the Ministry of Health (DHIS2, Oslo, Norway).

### Data analysis

We compared patient characteristics between pre- and post-intervention periods using chi-squared and Fisher’s exact tests for proportions and the Wilcoxon-Mann-Whitney *U* test for medians. Adjusting for site as a fixed effect, we used Poisson models with robust standard errors to contrast pre- and post-intervention rates of measuring vital signs, then calculated marginal rate differences with 95% confidence intervals (CI) using standardization [18]. We used the same approach to estimate the effects of period on severe illness diagnosis and in-hospital mortality, as well as the effects of severe illness conditions on mortality, controlling for period. We explored site-time interactions in each of the models. We conducted all analyses using Stata (Release 14, College Station, TX, USA).

## Results

### Study population characteristics

A total of 5865 patients were admitted to medical wards across all four health facilities between August 1, 2014, and May 31, 2015. We included 5759 patients and excluded 106 because either the date of admission was unknown or because the patient was under 14 years of age. Of the 5759 eligible patients, 1633 (28.9%) were admitted during the pre-intervention period and 4126 (71.1%) during the intervention period (Additional file 1: Figure S3). Demographic and clinical characteristics were similar between patients admitted during the pre- and post-intervention periods, except for HIV serostatus (Table 2).

**Table 2:** Clinical and demographic characteristics of study participants

Patient characteristic *n* (%)^a^	Pre-intervention period *n* = 1633	Intervention period *n* = 4126	*P* value
Male sex^b^	669 (41.1)	1755 (42.6)	0.31
Median age, years^c^ (IQR)	38 (24–55)	37 (23–58)	0.95
HIV-seropositive^d^	116 (20.7)	279 (14.8)	0.001
Admitting diagnosis^e^			
Malaria	527 (34.1)	1451 (37.0)	0.05
Peptic ulcer disease	161 (10.1)	445 (11.3)	0.33
Severe hypertension	125 (8.1)	265 (6.8)	0.08
Diabetic crisis	58 (3.8)	188 (4.8)	0.09
Anemia	75 (4.9)	156 (4.0)	0.14
Pneumonia/LRTI	72 (4.7)	168 (4.3)	0.53
CHF	61 (4.0)	136 (3.5)	0.39
Urinary tract infection	54 (3.5)	195 (5.0)	0.02
Other^f^	413 (26.7)	924 (23.4)	0.02
Median length of stay, days (IQR)	3 (2–6)	3 (2–6)	0.06

*Abbreviations*: *CHF* congestive heart failure, *IQR* interquartile range, *LRTI* lower respiratory tract infection

^a^Unless otherwise specified

^b^Missing in 12 patients

^c^Missing in 42 patients

^d^Assessed based on chart documentation of known history of HIV infection and/or results of rapid diagnostic or laboratory testing; missing in 3308 patients

^e^Missing in 285 patients

^f^Includes gastroenteritis, pelvic inflammatory disease, tuberculosis, and asthma/chronic obstructive pulmonary disease. Admitting diagnosis determined by admitting clinician

### SIMS implementation

The SIMS platform was implemented across the study sites generally as designed, with a few adaptations (Table 1). Designed to occur twice monthly, collaborative improvement meetings occurred every 2–4 weeks at each site. Clinical mentoring sessions, designed to occur monthly, occurred every 4–6 months at each site. Audit and feedback reports and text messages were implemented as designed.

### Vital sign collection

**Fig. 1:**
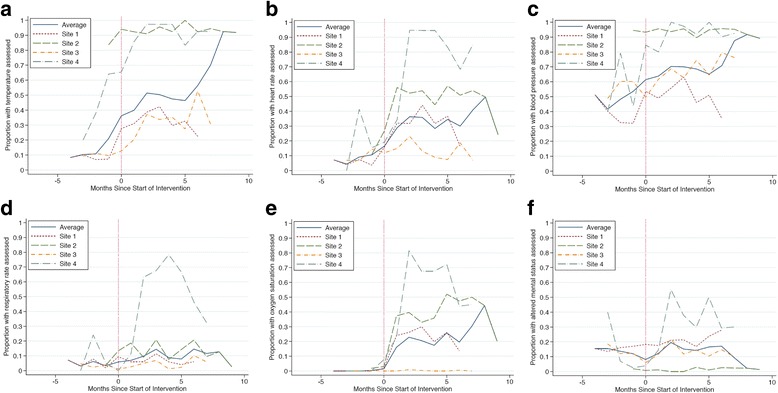
Changes in vital sign collection over study period, stratified by health facility. **a** Temperature. **b** Heart rate. **c** Blood pressure. **d** Respiratory rate. **e** Oxygen saturation. **f** Mental status. “Zero” on horizontal axis refers to time of initiation of SIMS intervention

Compared to the pre-intervention period, there were significant increases in clinician assessment of all vital signs (temperature, heart rate, respiratory rate, blood pressure, pulse oximetry, and mental status) during the intervention period (Table 3). We observed marked improvements in assessment of temperature, heart rate, blood pressure, and pulse oximetry across nearly all facilities, while improvements in respiratory rate and mental status assessment were less consistent and less sustained (Fig. 1, Additional file 1: Table S4a). Health facility 4 registered the greatest gains and the highest end performance on all indicators. Health facility 2 improved markedly in assessing heart rate but did not show significant improvement in assessment of mental status, temperature, or blood pressure, although the high rates at which clinicians measured temperature and blood pressure during the pre-intervention period left minimal room for improvement in these processes (Additional file 1: Table S4b). For all indicators except mental status, the absolute final performance level was lower for health facilities 1 and 3 than for health facilities 2 and 4.

**Table 3:** Impact of SIMS intervention on collection of vital signs (adjusted for facility; for site-level estimates, see Tables S4a and S4b)

Vital sign % (95% CI)	Pre-intervention period *n* = 1633	Intervention period *n* = 4126	Difference	*P* value
Temperature	21 (9–34)	48 (44–52)	+ 27 (+11 to +43)	0.001
Heart rate	10 (3–17)	32 (29–34)	+ 22 (+12 to +32)	< 0.001
Blood pressure	54 (49–59)	69 (67–70)	+ 15 (+8 to +21)	< 0.001
Respiratory rate	5 (3–7)	10 (9–11)	+ 5 (+2 to +8)	0.002
Pulse oximetry	0.2 (0.0–0.6)	19 (19–20)	+ 19 (+19 to +20)	< 0.001
Mental status	11 (9–13)	15 (14–16)	+ 4 (+2 to +7)	0.002

*Abbreviations*: *CI* confidence Interval, *SIMS* Severe Illness Management System

### Diagnosis of severe illness conditions

Among all enrolled patients, 21.5% (1236/5579) met criteria for severe illness. Specifically, 15.2% (875/5579) met criteria for shock, 3.4% (195/5759) for sepsis (195/5759), 3.2% (185/5759) for severe respiratory distress, and 4.1% (234/5759) for altered consciousness. Compared to the pre-intervention period, patients admitted during the intervention period were significantly more likely to be diagnosed with sepsis (0.4 vs. 4.0%, risk ratio (RR) 10.1, 95% CI 3.3–30.7, *p* = 0.001) and severe respiratory distress (0.9 vs. 3.9%, RR 4.5, 95% CI 1.8–10.9, *p* = 0.001) (Table 4). We also found a trend toward patients in the intervention period being substantially more likely to be diagnosed with shock (10.8 vs. 16.7%, RR 1.5, 95% CI 0.9–2.5, *p* = 0.09).

**Table 4:** Impact of SIMS intervention on diagnosis of severe illness conditions (adjusted for site)

Severe illness condition % (95% CI)	Pre-intervention period *n* = 1633	Intervention period *n* = 4126	Risk ratio	*P* value
Sepsis	0.4 (0.0 to 0.9)	4.3 (4.2–4.5)	10.1 (3.3–30.7)	0.001
Severe respiratory distress	0.9 (0.1–1.6)	3.9 (3.7–4.1)	4.5 (1.8–10.9)	0.001
Shock	10.8 (6.4–15.2)	16.7 (15.1–18.2)	1.5 (0.9–2.5)	0.09
Altered mental status^a^	5.2 (4.7–5.7)	3.5 (3.3–3.7)	0.7 (0.6–0.8)	<0.001

*Abbreviations*: *CI* confidence interval, *SIMS* Severe Illness Management System

^a^Altered mental status defined as anything less than alert on the AVPU scale (alert (A), responsive to verbal stimuli (V), responsive to painful stimuli (P), and unresponsive (U))

### In-hospital mortality

Among 5272 (90%) patients with available data on vital status, 203 (3.9%) died prior to hospital discharge. When adjusted for study site, patients meeting criteria for severe illness conditions were significantly more likely to die prior to hospital discharge (7.4 vs. 2.9%; RR 2.6, 95% CI 2.4–2.7, *p* < 0.001). In the adjusted analyses, patients with shock were significantly more likely to die prior to discharge compared to patients without shock (6.3 vs. 3.4%, RR 1.9, 95% CI 1.5–2.3, *p* < 0.001). Patients with severe respiratory distress were also more likely to die than those without severe respiratory distress (15.6 vs. 3.4%, RR 4.5, 95% CI 3.8–5.4, *p* < 0.001), as were patients with altered mental status compared to those without altered mental status (11.5 vs. 3.5%, RR 3.25, 95% CI 2.5–4.2, *p* < 0.001). There was no increased risk of in-hospital death for patients with sepsis compared to those without sepsis (3.0 vs. 3.9%; RR 0.8, 95% CI 0.45–1.3, *p* = 0.32). Mortality rates were similar in the intervention and pre-intervention periods (3.7 vs. 4.3%, RR 0.8, 95% CI 0.6–1.3, *p* = 0.47).

## Discussion

In this study, we assessed a multi-modal intervention to improve implementation of WHO guidelines for vital sign collection and diagnosis of severe illness among hospitalized adolescents and adults in western Uganda. Our results suggest that severe illness is under-recognized and associated with substantial mortality in public and private community hospitals in a rural district of Uganda. After introducing the SIMS platform, we observed significant improvements in vital sign collection and diagnosis of key severe illness conditions, although overall rates of vital sign collection remained sub-optimal. Similar theory-informed, multi-modal implementation studies have been conducted to improve care for severely ill children in low-income settings [8]. However, to our knowledge, this is among the first to evaluate such an approach in severely ill adults and adolescents.

We found that by the end of the intervention period, nearly one in four admitted patients had met triage criteria for one or more severe illness conditions. Our estimates are probably conservative because more complete collection of vital signs would likely identify additional patients with severe illness. Although limited compared to high-income settings, available data suggest that a large percentage of death and disability in low-income settings is the result of acute, severe illness [19, 20]. This is particularly the case in high HIV-burden settings in Sub-Saharan Africa similar to ours, where severe opportunistic and non-opportunistic infections are leading causes of hospitalization and death [21]. Historically, investments in health care services in Sub-Saharan Africa have focused on bolstering outpatient care, especially delivery of preventive services to pregnant women and children and anti-retroviral therapy to HIV-infected individuals [20, 22, 23]. Given the high burden of HIV-associated severe illness in the region and the potential benefits of basic supportive interventions for acutely ill patients, improvements in the capacity to provide basic emergency care to all populations are urgently needed. Such improvements may also enhance community confidence in local health systems, thereby facilitating engagement in preventive services and reducing delays in presentation to care [24–26].

We used the vital sign-derived WHO “Quick Check” tool to identify severe illness in a low-income setting. In-hospital mortality was significantly higher among patients meeting these criteria for severe illness, with over 7% dying in the hospital. In low-income settings, the majority of severely ill patients receive care on general hospital wards not only because of a lack of intensive care facilities but also because severe illness is under-recognized [3, 4]. Thus, improved early recognition and supportive management of patients with clinical decompensation are essential in low-income settings. Although little is known about the utility of triage tools based on physiologic parameters in low-income settings, available data suggest that such tools can identify hospitalized patients at risk for deterioration and death from sepsis and other severe illness conditions [27, 28]. For example, recent studies from Uganda and Tanzania found that patients meeting criteria for severe illness based on physiologic parameters measured at bedside were significantly more likely to die in the hospital [27, 29]. Given the low cost of Quick Check, its substantial incremental value for diagnosing severe illness in this study, and the demonstrated value of similar bedside triage tools for identifying severely ill patients before they progress to organ failure and death, additional studies are needed to further validate its prognostic utility [3, 20, 30].

In this study, we observed low frequencies of vital sign collection following the WHO Quick Check + training program and before the SIMS intervention. Although conflicting data exist about whether or not multi-modal interventions are more effective than single-component interventions [31–34], in the context of our SIMS intervention incorporating collaborative improvement meetings, clinical performance audits and feedback, and clinical mentoring, we observed significant improvements in vital sign collection and severe illness diagnoses. Similar interventions have yielded significant improvements in management of severely ill children in Sub-Saharan Africa and obstetric conditions in Latin America and show the promise of theory-informed multi-modal approaches to improve adherence to a range of other clinical practice guidelines [8, 35–37].

Although the SIMS intervention was implemented generally as designed, several challenges existed to delivering the expected dose of the intervention, especially for clinical mentoring sessions. This was related primarily to logistical challenges in scheduling travel by expert clinicians to rural study sites from the capital city of Kampala. Future iterations of SIMS and similar interventions would benefit from a regionally based pool of expert clinicians to deliver mentoring and from leveraging telehealth to provide clinical mentoring through virtual communities of practice [38, 39].

Although we observed an improvement in vital sign collection during the study period, this improvement varied across specific vital signs, with the least improvement observed for respiratory rate and mental status. During collaborative improvement meetings, clinicians reported that a lack of functioning wall clocks and wrist watches hindered measurement of respiratory rates. Future interventions would benefit from ensuring the availability of these simple yet important tools for clinical care. Second, many clinicians reported documenting vital signs, especially mental status, only when these findings were abnormal and reported that they favored an alternative clinical tool (the Glasgow Coma Scale) for mental status assessment to the one recommended in Quick Check + (the Alert Voice Pain Responsive (AVPU) scale), although the latter has been shown to be simpler, faster, and more reproducible [40, 41]. Future interventions should consider the need for longer periods of implementation to allow for additional detailing of providers, acceptance of new tools, and acquisition of new skills.

We observed that the effects of SIMS on vital sign collection declined approximately halfway through the study period, with overall rates of vital sign collection remaining sub-optimal, particularly at the two public hospital study facilities. There are several potential reasons for these observations. First, when faced with human resource limitations, health facility staff, particularly at the public facilities with lower staffing ratios, reported prioritizing empiric treatment interventions (intravenous fluids, antimicrobials) over collection of vital signs, which they believed were less important to guide clinical care. Next, although SIMS provided funding for the purchase of instruments for measuring vital signs, these items were often lost or broken as the study period progressed, with facility staff reporting difficulties in repairing or purchasing new equipment. Last, as mentioned above, many clinicians reported documenting vital signs only if they were abnormal. Future interventions must emphasize the importance of consistent clinical documentation to facilitate routine quality assurance and monitoring. Furthermore, these findings emphasize that the success of interventions to change behavior often depends on the availability and sustainability of an adequate physical environment; in this case, the supplies and equipment needed to perform the target behavior.

Inpatient mortality was lower among our patient population than the 33–60% reported from large studies of sepsis and acute respiratory distress syndrome in Sub-Saharan Africa [11, 42–44]. This likely reflects differences in study settings and mortality denominators, as our cohort was comprised of consecutive patients admitted to rural, general hospitals, as opposed to those admitted to urban referral hospitals with severe illness identified at enrollment [42–44]. We also defined severe illness based on the WHO Quick Check triage criteria which seek to maximize sensitivity for detection at the cost of some overdiagnosis. Nevertheless, the Quick Check definitions of severe illness conditions effectively identified syndromic sub-groups with substantially increased risk of mortality. Although we did not observe an improvement in mortality across study periods, there are multiple variables beyond vital sign collection that are likely to contribute to inpatient outcomes for severely ill patients in low-income settings. These include early resuscitation interventions (prompt administration of effective antimicrobials, supplemental oxygen, and fluid resuscitation) and host factors (time to hospital presentation and illness severity at hospital presentation). Because the SIMS intervention was designed primarily to improve vital sign collection, future studies are needed to improve implementation of resuscitative interventions for severely ill patients and identify patient populations in whom such interventions are likely to be most beneficial.

This study has multiple strengths. First, we provide a large, prospective description of the burden of severe illness in a low-income setting. Drawing on a large dataset from multiple primary level health facilities, our results identify a high frequency of severe illness in a high HIV-prevalence setting and provide real-world performance data on the diagnosis of severe illness conditions in rural hospitals. Second, our study highlights existing challenges to optimal care for severely ill hospitalized patients in low-income settings, specifically poor triage and vital sign collection practices, limited availability of basic equipment for measuring and monitoring vital signs, and poor clinical documentation practice.

This study also has several potential limitations. First, improvements in vital sign collection and diagnosis could be attributed to underlying secular trends that may have resulted in our inaccurately attributing improvements in vital sign collection and severe illness diagnoses to our intervention. However, we noted the greatest performance gains in the months immediately after the interventions were introduced, which makes a simple “observer” effect a less likely explanation for the improvements we observed [45]. Second, our selection of study sites based on the availability of logistical support for training and ongoing quality improvement activities may have resulted in better opportunities for a positive response to the SIMS intervention. Although performance increased for all vital signs, improvements remained sub-optimal and increases varied by individual vital signs and across sites, with the two lowest performing sites (health facilities 1 and 3) being public hospitals with higher patient volumes and lower staffing ratios. As the SIMS intervention was rolled out in a consistent format at each site, the observed variations in implementation reinforce the need for continuous assessment of context-specific barriers to foster sustained improvements in implementation, as well as the substantial and at times irrevocable barriers presented by human and material resource constraints. Third, we lack detailed data about which elements of our multi-component SIMS intervention had the greatest effects in augmenting performance or about whether all were necessary. Future evaluations are necessary to collect such data and assess the impact of individual components of our intervention. Finally, although we did not quantify costs of the setting up and maintaining the SIMS platform, our intervention required substantial study staff time, and we provided considerable monetary support to study facilities. Future studies examining the effects of the multi-modal SIMS intervention on clinical endpoints should also measure the ratio of costs to benefits to inform scalability. Specifically, they should compare the incremental value of SIMS and other post-training interventions to training alone. In addition, they should determine if setup costs can be affordably distributed across sites and maintenance costs absorbed by quality improvement programs that already exist within the Ministry of Health and local health facilities.

## Conclusions

Severe illness is under-recognized and associated with substantial mortality in western Uganda. Our innovative, multi-modal quality improvement intervention enhanced vital sign collection and diagnosis of severe illness according to WHO guidelines and represents a promising platform to strengthen management of severe illness and adherence to other clinical practice guidelines. Further studies are needed to evaluate the impact of similar interventions on severe illness care in low-income settings.

## Additional file

Additional file 1: Appendix S1. Pre-training facility assessment tool. **Appendix S2.** Quick Check + hospital assessment report of existing severe illness practices. **Table S1.** Characteristics of inpatient health facilities participating in SIMS intervention. **Table S2.** Diagnostic criteria for severe illness conditions covered in Quick Check + training program, as defined by the World Health Organization District Clinician Manual. **Table S3a.** Barriers to executing target behaviors*, as documented by hospital staff while formulating implementation plans and placed into COM-B domains. **Table S3b.** Intervention functions targeting identified barriers and facilitators as defined in the Behavioral Change Wheel framework. Table S4a Between-site variation in vital sign collection before and after SIMS introduction, by site. **Table S4b.** Between-site variation in impact of SIMS on vital sign collection, by vital sign. **Figure S1.** Diagram illustrating the conceptual model based on components of the COM-B model that was utilized to develop the SIMS intervention. **Figure S2.** Staggered, pre-post quasi-experimental study design utilized for implementation of SIMS intervention. Baseline period indicates time period following Quick Check + training and before SIMS intervention. Intervention period indicates time period during which SIMS intervention was implemented. **Figure S3.** Flow diagram for patients included in study (DOCX 607 kb)

## Abbreviations

CI: Confidence interval
IMAI: Integrated Management of Adolescent and Adult Illness
RR: Risk ratio
SIMS: Severe Illness Management System
WHO: World Health Organization

## References

[1] Adhikari NKJ, Fowler RA, Bhagwanjee S, Rubenfeld GD (2010). Critical care and the global burden of critical illness in adults. Lancet.

[2] Bhagwanjee S (2006). Critical care in Africa. Crit Care Clin.

[3] Murthy S, Adhikari NK (2013). Global health care of the critically ill in low-resource settings. Ann Am Thorac Soc..

[4] Albert TJ, Fassier T, Chhuoy M, Bounchan Y, Tan S, Ku N (2015). Bolstering medical education to enhance critical care capacity in Cambodia. Ann Am Thorac Soc.

[5] World Health Organization. IMAI district clinician manual: hospital care for adolescents and adults. World Health Organization. 2011. http://www.who.int/hiv/pub/imai/imai2011/en/. Accessed 29 April 2017.

[6] Grol R, Grimshaw J (1999). Evidence-based implementation of evidence-based medicine. Jt Comm J Qual Improv.

[7] Prior M, Guerin M, Grimmer-Somers K (2008). The effectiveness of clinical guideline implementation strategies––a synthesis of systematic review findings. J Eval Clin Pract.

[8] Ayieko P, Ntoburi S, Wagai J, Opondo C, Opiyo N, Migiro S (2011). A multifaceted intervention to implement guidelines and improve admission paediatric care in Kenyan district hospitals: a cluster randomised trial. PLoS Med.

[9] Uganda Bureau of Statistics. National Population and Housing Census 2014. Kampala: Uganda Bureau of Statistics. http://www.ubos.org/2016/03/24/census-2014-final-results/. Accessed 29 Mar 2017.

[10] Michie S, van Stralen MM, West R. The behaviour change wheel: a new method for characterising and designing behaviour change interventions. Implement Sci 2011;6:42. 10.1186/1748-5908-6-42 PMC3096582 21513547

[11] Michie S, Atkins L, West R (2014). The behaviour change wheel: a guide to designing interventions.

[12] Ayakaka I, Ackerman S, Ggita JM, Kajubi P, Dowdy D, Haberer JE, Fair E (2017). Identifying barriers to and facilitators of tuberculosis contact investigation in Kampala, Uganda: a behavioral approach. Implement Sci.

[13] Jacob ST, Banura P, Baeten JM, Moore CC, Meya D, Nakiyingi L (2012). The impact of early monitored management on survival in hospitalized adult Ugandan patients with severe sepsis: a prospective intervention study. Crit Care Med.

[14] Asiimwe SB, Okello S, Moore CC (2014). Frequency of vital signs monitoring and its association with mortality among adults with severe sepsis admitted to a general medical ward in Uganda. PLoS One.

[15] Cummings MJ, Wamala JF, Bakamutumaho B, Davis JL (2016). Vital signs: the first step in prevention and management of critical illness in resource-limited settings. Intensive Care Med.

[16] Nilsen P (2015). Making sense of implementation theories, models and frameworks. Implement Sci.

[17] Deming WE (1993). The new economics.

[18] Zou G (2004). A modified Poisson approach to prospective studies with binary data. Am J Epidemiol.

[19] Obermeyer Z, Abujaber S, Makar M, Stoll S, Kayden SR, Wallis LA (2015). Emergency care in 59 low- and middle-income countries: a systematic review. Bull World Health Organ.

[20] Razzak JA, Kellermann AL (2002). Emergency medical care in developing countries: is it worthwhile?. Bull World Health Organ.

[21] Ford N, Shubber Z, Meintjes G, Grinsztejn B, Eholie S, Mills EJ (2015). Causes of hospital admission among people living with HIV worldwide: a systematic review and meta-analysis. Lancet HIV.

[22] Hirshon JM, Risko N, Calvello EJ, Stewart de Ramirez S, Narayan M, Theodosis C (2013). Health systems and services: the role of acute care. Bull World Health Organ.

[23] Lawn SD, Harries AD, Anglaret X, Myer L, Wood R (2008). Early mortality among adults accessing antiretroviral treatment programmes in sub-Saharan Africa. AIDS.

[24] Molyneux E, Ahmad S, Robertson A (2006). Improved triage and emergency care for children reduces inpatient mortality in a resource-constrained setting. Bull World Health Organ.

[25] Riviello ED, Letchford S, Achieng L, Newton MW (2011). Critical care in resource-poor settings: lessons learned and future directions. Crit Care Med.

[26] Moresky RT, Bisanzo M, Rubenstein BL, Hubbard SJ, Cohen H, Ouyang H (2013). A research agenda for acute care services delivery in low- and middle-income countries. Acad Emerg Med.

[27] Kruisselbrink R, Kwizera A, Crowther M, Fox-Robichaud A, O'Shea T, Nakibuuka J (2016). Modified early warning score (MEWS) identifies critical illness among ward patients in a resource restricted setting in Kampala, Uganda: a prospective observational study. PloS One.

[28] Asiimwe SB, Abdallah A, Ssekitoleko R. A simple prognostic index based on admission vital signs data among patients with sepsis in a resource-limited setting. Crit Care. 2015;19:86. 10.1186/s13054-015-0826-8 PMC4360926 25888322

[29] Baker T, Blixt J, Lugazia E, Schell CO, Mulungu M, Milton A (2015). Single deranged physiologic parameters are associated with mortality in a low-income country. Crit Care Med.

[30] Churpek MM, Snyder A, Han X, Sokol S, Pettit N, Howell MD (2017). Quick Sepsis-related Organ Failure Assessment, Systemic Inflammatory Response Syndrome, and early warning scores for detecting clinical deterioration in infected patients outside the intensive care unit. Am J Respir Crit Care Med.

[31] English M, Ntoburi S, Wagai J, Mbindyo P, Opiyo N, Ayieko P (2009). An intervention to improve paediatric and newborn care in Kenyan district hospitals: understanding the context. Implement Sci.

[32] English M, Nzinga J, Mbindyo P, Ayieko P, Irimu G, Mbaabu L (2011). Explaining the effects of a multifaceted intervention to improve inpatient care in rural Kenyan hospitals––interpretation based on retrospective examination of data from participant observation, quantitative and qualitative studies. Implement Sci.

[33] NHS Centre for Reviews and Dissemination. Effective health care: getting evidence into practice. Royal Society of Medicine. 1999: https://www.york.ac.uk/media/crd/ehc51.pdf. Accessed 12 Apr 2017.

[34] Squires JE, Sullivan K, Eccles MP, Worswick J, Grimshaw JM (2014). Are multifaceted interventions more effective than single-component interventions in changing health-care professionals’ behaviours? An overview of systematic reviews. Implement Sci.

[35] English M (2013). Designing a theory-informed, contextually appropriate intervention strategy to improve delivery of paediatric services in Kenyan hospitals. Implement Sci.

[36] Baker T, Schell CO, Lugazia E, Blixt J, Mulungu M, Castegren M (2015). Vital signs directed therapy: improving care in an intensive care unit in a low-income country. PLoS One.

[37] Althabe F, Buekens P, Bergel E, Belizán JM, Campbell MK, Moss N (2008). A behavioral intervention to improve obstetrical care. N Engl J Med.

[38] Arora S, Thornton K, Murata G, Deming P, Kalishman S, Dion D (2011). Outcomes of treatment for hepatitis C virus infection by primary care providers. N Engl J Med.

[39] Walsh K (2014). Virtual communities of practice: overcoming barriers of time and technology. J Med Internet Res.

[40] Kelly CA, Upex A, Bateman DN (2004). Comparison of consciousness level assessment in the poisoned patient using the alert/verbal/painful/unresponsive scale and the Glasgow Coma Scale. Ann Emerg Med.

[41] McNarry AF, Goldhill DR (2004). Simple bedside assessment of level of consciousness: comparison of two simple assessment scales with the Glasgow Coma Scale. Anaesthesia.

[42] Jacob ST, Moore CC, Banura P, Pinkerton R, Meya D, Opendi P (2009). Severe sepsis in two Ugandan hospitals: a prospective observational study of management and outcomes in a predominantly HIV-1 infected population. PLoS One.

[43] Riviello ED, Kiviri W, Twagirumugabe T, Mueller A, Banner-Goodspeed VM, Officer L (2016). Hospital incidence and outcomes of the acute respiratory distress syndrome using the Kigali modification of the Berlin definition. Am J Respir Crit Care Med.

[44] Andrews B, Muchemwa L, Kelly P, Lakhi S, Heimburger DC, Bernard GR (2014). Simplified severe sepsis protocol: a randomized controlled trial of modified early goal-directed therapy in Zambia. Crit Care Med.

[45] McCarney R, Warner J, Iliffe S, van Haselen R, Griffin M, Fisher P. The Hawthorne Effect: a randomised, controlled trial. BMC Med Res Methodol 2007;7:30. 10.1186/1471-2288-7-30 PMC1936999 17608932

