# Supplementary material for: A complex intervention to improve implementation of World Health Organization guidelines for diagnosis of severe illness in low-income settings: a quasi-experimental study from Uganda
Source: Implement Sci. 2017 Nov 6;12:126. doi: 10.1186/s13012-017-0654-0 (PMC5674818; doi:10.1186/s13012-017-0654-0)
Supplement: Additional file 1: Appendix S1. — Pre-training facility assessment tool. Appendix S2. Quick Check + hospital assessment report of existing severe illness practices. Table S1. Characteristics of inpatient health facilities participating in SIMS intervention. Table S2. Diagnostic criteria for severe illness conditions covered in Quick Check + training program, as defined by the World Health Organization District Clinician Manual. Table S3a. Barriers to executing target behaviors*, as documented by hospital staff while formulating implementation plans and placed into COM-B domains. Table S3b. Intervention functions targeting identified barriers and facilitators as defined in the Behavioral Change Wheel framework. Table S4a Between-site variation in vital sign collection before and after SIMS introduction, by site. Table S4b. Between-site variation in impact of SIMS on vital sign collection, by vital sign. Figure S1. Diagram illustrating the conceptual model based on components of the COM-B model that was utilized to develop the SIMS intervention. Figure S2. Staggered, pre-post quasi-experimental study design utilized for implementation of SIMS intervention. Baseline period indicates time period following Quick Check + training and before SIMS intervention. Intervention period indicates time period during which SIMS intervention was implemented. Figure S3. Flow diagram for patients included in study (DOCX 607 kb) [file 13012_2017_654_MOESM1_ESM.docx]

**Additional File**

**Title**: A complex intervention to improve implementation of World Health Organization guidelines for diagnosis of severe illness in low-income settings: a quasi-experimental study from Uganda

**Authors:**

Matthew J. Cummings, MD, Elijah Goldberg, BA, Savio Mwaka, BA, Olive Kabajaasi, BA, Eric Vittinghoff, PhD, Adithya Cattamanchi, MD, MAS, Achilles Katamba, MBChB, PhD, Nathan Kenya-Mugisha, MBChB, MMed, Shevin T. Jacob, MD, MPH, and J. Lucian Davis, MD, MAS

**Additional File - Appendix**

**Appendix S1. Pre-Training Facility Assessment Tool.**

**HOSPITAL SURVEY PRIOR TO QUICK CHECK/SEVERELY ILL PATIENT TRAINING**

Please answer the following questions regarding resources in you casualty area. By **casualty area**, we are referring to the place where medical emergency or casualty patients are treated (often called the emergency room) OR to the outpatient department if there is no casualty area at your hospital or health centre IV.

Completion of this survey is voluntary. Results will be discussed during the upcoming training with the team from this hospital.

**Patient flow for adults and adolescents arriving with emergencies**

**Source**: Visit to the emergency department/casualty and interviews with staff dealing with emergencies.

**Instructions:** Interview staff where emergencies present, who would see them; how senior staff are called, and where and how severe conditions are handled.

10. Where are non pregnant adolescent and adult patients with an emergency (medical or surgical) condition received?

…………………………………………………………………………….………………………………………………..

…………………………………………………………………………….………………………………………………..

11. Describe adult patient flow of a typical emergency (patients presenting as an emergency to hospital):

………………………………………………………………………………………………………………………………

…………………………………………………………………………….……………….............................................

12. Are adults/adolescents assessed for severity/ priority signs (triaged) immediately on arrival? Y □ N □

13. Do sick adult patients have to wait for their turn, registration, payment etc. before a first assessment is done and action taken? Y □ N □

14. Is there any system in place to prioritize the most severely ill adults and adolescents(triage)?Y □ N □

If so, describe: .………………………………………………………………………………………………………..

…………………………………………………………………………………………………………………………..

…………………………………………………………………………………….…………………………………….

15. Is there an emergency management area equipped to take care of severely ill adults/ adolescents? Y□ N □

If so, describe: .…………………………………………………………………………………………………………

……………………………………………………………………………………………………………………………

…………………………………………………………………………………….……………………………………..

16. Is there a separate consultation area for moderately ill adults with priority signs? Y □ N □

If so, describe: .…………………………………………………………………………………………………………

………...………………………………………………………………………………………………………………….

17. Is this separate from the normal outpatient facility dealing with non-emergency patients? Y □ N □

If so, describe: .………………………………………………………………………………………….

…………………………………………………………………………………………………………….

18. How are severely ill patients diagnosed and handled in the outpatient department (i.e. patients presenting normally to the outpatient department, but severely ill)?

Describe patient flow. ….………………………………………………………………………………………………..

……………………………………………………………………………………………………………………………..

…………………………………………………………………………………….………………………………………..

19. Are emergencies in pregnant women dealt with in a separate area to general ER? Y □ N □

If so, describe...............................................................................................................................

......................................................................................................................................................

20. Are emergencies in young children dealt with in a separate area to the general ER/casualty? Y □ N □

21. Do patients come with referral notes when they have been referred from first level units?

Never □ Sometimes □ Always □

Comments: .…………………………………………………………………………….………………….

………...……………………………………………………………………………………………………..

22. Are there any job aids (wall charts, chart booklets) displayed for identifying adults by severity of condition and for management of adult emergencie? Y □ N □

If so, describe about what, and comment on adequacy:……………………………………………………..

………………..…………………………………………………………………………………………………….

………...……………………………………………………………………………………………………………

23. Distance from reception area to emergency management area:

In the same building, distance....................................................................................................................

In another building, distance.......................................................................................................................

24. Distance from consultation area to emergency management area:

In the same building, distance....................................................................................................................

In another building, distance.......................................................................................................................

25. Is there a system in place to call for help within the facility in emergencies? Y □ N □

If so, describe.............................................................................................................................

Time to arrive (if not at home) ....................................

26. Are the staff who are trained in emergencies [ ] On site/24hrs [ ] On site some of the time ; otherwise at home on call [ ] On site some of the time, otherwise not available

27. Does this facility have a functioning landline telephone to call outside? Y □ N □

If yes, is it available at all times?

28. Does this facility have a functioning cell phone supported by the facility? Y □ N □

If no, do health workers use their own cell phones to call out for consultation or help in emergencies? Y □ N □

29. Does this facililty have a functioning short-wave radio? Y □ N □

30. Does this facility have a functioning computer with internet access? Y □ N □

If yes, is it working today? Y □ N □

**CASUALTY AREA (EMERGENCY ROOM) TODAY (plus review of fluid availability)**

31. Today in the casualty area, how many of each healthcare worker are working and how many have been trained on adult Quick Check or paediatric ETAT? WRITE the number in each space.

a. medical officers ____ ; of whom _____ have been trained in Quick Check; of whom___ trained in ETAT

b. clinical officers ____ ; of whom _____ have been trained in Quick Check; of whom___trained in ETAT

c. nurses ____ ; of whom _____ have been trained in Quick Check; of whom___trained in ETAT

32. Today in the casualty area, are adolescent and adult patients being triaged using the Quick Check (Emergency-Priority-Que) system? CHECK one response. [ ] YES [ ] NO

33. Today in the casualty area, are paediatric patients being triaged using ETAT?

CHECK one response. [ ] YES [ ] NO

34. Today, how much intravenous fluid -Lactated Ringer’s or 0.9% Normal Saline- is available to treat patients? CHECK one response for each area

In the casualty area [ ] 0-4 L [ ] 5-10 L [ ] 11-15 L [ ] 16-20 L [ ] >20 L

On the adult ward [ ] 0-4 L [ ] 5-10 L [ ] 11-15 L [ ] 16-20 L [ ] >20 L

Then fill in number of litres:

What is the daily ration for the casualty area? …… L

What is the daily ration for the adult ward?……….L

What is the daily ration for the whole hospital?………L What is the monthly ration for the whole hospital?.........L

35. Today in the casualty area, which of the following treatments are available to treat patients? CHECK each available treatment.

[ ] Ceftriaxone

[ ] Salbutamol- liquid for nebulizer

[ ] Salbutamol- metered-dose inhaler

[ ] 50% Dextrose

[ ] Adrenaline IM (Epinephrine)

[ ] Diazepam

[ ] Magnesium sulfate (IV)

[ ] Blood transfusion

[ ] Artsenuate IV or IM

[ ] Quinine IV or IM

[ ] Ampicillin

36. Today in the casualty area, which of the following oxygen supplies are available to treat patients? CHECK each available item.

[ ] Working oxygen cylinder

[ ] Working oxygen concentrator

[ ] Face mask with no reservoir bag

[ ] Face mask with reservoir bag (non-rebreather)

[ ] Nasal prongs

[ ] Oral airway

[ ] Bag-valve mask (adult size)

[ ] Bag-valve mask (pediatric size)

37. Today in the casualty area, which of the following available to monitor patients? CHECK each available item.

[ ] Blood pressure machine

[ ] Thermometer

[ ] Stethoscope

[ ] Pulse oximeter

[ ] Glucometer

38. Today in the casualty area, which of the following diagnostic tests are available? CHECK each available test

[ ] Haemoglobin or haematocrit

[ ] Type and crossmatch

[ ] Glucose

[ ] Rapid diagnostic test for malaria

[ ] Malaria smear

[ ] Urinalysis

[ ] Blood culture

[ ] Chest xray

[ ] Ultrasound

**ADULT WARDS**

39. Is there a separate ward for adults/adolescents? Y □ N □

(separate from children & maternity)

40. If yes, how many adult wards does this hospital have? ……..

41. If yes, are there separate female and male wards? Y □ N □

42. How many adult inpatient beds does this hospital have (non-maternity)?..............

43. Is there an Intensive Care Unit? Y □ N □ If yes, describe:…………………………….

44. On the ward, are the most seriously ill adults cared for in a section where they receive closest attention? Y □ N □

45. Is this section close to the nursing station so that these patients can be directly observed most of the time? Y □ N □

**Monitoring- adults and adolescents**

46. At the time of admission, is a monitoring plan prescribed according to the severity of the patient's condition? Y □ N □

47. Is a standard monitoring chart used with the following information: patient details; vital signs; clinical signs depending on condition; treatments given, feeding and outcome? Y □ N □

48. Are all patients reassessed daily during working days by a clinician? Y □ N □

49. Are seriously ill patients reassessed by a clinician upon admission and reviewed at least twice daily until improved? Y □ N □

50. Are key risk signs monitored and recorded by a nurse twice a day and at least eight times a day for critically ill patients? Y □ N □

51. Are doses and time recorded for medications and IV-fluids given by the nurse for every patient receiving medication or IV-fluids? Y □ N □

52. Is additional special monitoring performed and recorded appropriately when needed to follow the progress of particular conditions: e.g. for fluid balance (input – output) in severe respiratory distress, oxygen, coma scale for unconscious patients? Y □ N □

53. Do nurses use the results of patient monitoring to alert the physicians of problems or changing patient status warranting their attention? Y □ N □


**Appendix S2. Quick Check+ Hospital Assessment Report of Existing Severe Illness Practices**

*Hospital:*

*Location:*

*Date of visit:*

*Staff:*

*Prepared by:*

*Attachments:*

**Summary**

**Patient flow**

**Personnel**

**Oxygen availability**

**Vital signs monitoring equipment**

**Testing available**

**Patient monitoring**

**Infection control**

**Additional file - Tables**

**Table S1: Characteristics of inpatient health facilities participating in SIMS intervention**

| **Facility** | **Administration** | **Inpatient beds available** | **Inpatient admissions per year*** | **Outpatient visits per year*** |
| --- | --- | --- | --- | --- |
| Health Facility 1 | MoH | 110 | 13,783 | 72,110 |
| Health Facility 2 | Private charity | 272 | 8,814 | 47,094 |
| Health Facility 3 | MoH and private charity | 205 | 10,851 | 24,645 |
| Health Facility 4 | Private charity | 90 | 3,565 | 10,123 |

**Abbreviation:** MoH, Ministry of Health.

**Legend:** *Representative of data from 2013-2014

**Table S2: Diagnostic criteria for severe illness conditions covered in Quick Check+ training program, as defined by the World Health Organization District Clinician Manual**

| **Condition** | **Diagnostic criteria** |
| --- | --- |
| Shock | Systolic blood pressure ≤90 mm Hg *OR* heart rate ≥110 beats/minute |
| Sepsis | Presumed infection (based on admission diagnosis*^*^*)  *AND*  At least one of the following:  respiratory rate ≥24 breaths/minute,  heart rate ≥100 beats/minute,  temperature ≤36 or ≥38° Celsius |
| Severe respiratory distress | SpO_2_ <90% *OR* respiratory rate >30 breaths/min |
| Altered consciousness | Abnormal AVPU score:  responds to voice (V), responds to pain (P), or unresponsive (U) |

**Abbreviations:** SpO2, oxyhemoglobin saturation by pulse oximetry; AVPU: alert, voice, pain, unresponsive.

**Legend:** ^*^Presumed infection based on clinician admitting diagnoses of: amoebiasis, cholecystitis, colitis, diverticulitis, esophagitis, gastroenteritis, malaria, meningitis, pelvic inflammatory disease, pneumonia, tuberculosis, urinary tract infection.

**Table S3a: Barriers to executing target behaviors*, as documented by hospital staff while formulating implementation plans and placed into COM-B domains.**

|  | **Health Facility 1** | **Health Facility 2** | **Health Facility 3** | **Health Facility 4** |
| --- | --- | --- | --- | --- |
| **Capability** | “Inadequate skills by staff” | “Not all nurses can recognize emergency signs” | “Stock is often kept away because there [sic] value is not known” | “Knowledge gap in emergency handling” |
|  | “Inadequate information and education” | “Vital observations for the critically ill are not taken according to the set standards” |  |  |
| **Opportunity** | “High [numbers of] clientele” | “Only nurses are responsible for triaging” | “Equipment missing” | “At times stock outs of emergency drugs” |
|  | “Inadequate equipment” | “Inadequate stocks of emergency treatments” | “[Lack of] clinician meetings…have paralyzed the activities” | “No severely ill monitoring tools” |
|  | “No designated Emergency Room” | “Inappropriate response to nurses by some clinicians” | “Understaffing” | “Severely ill not near nursing station” |
|  | “Inadequate stationery [for clinical documentation]” |  |  |  |
|  | “Unclear patient triage process at night” |  |  |  |
|  | “Inadequate communication” |  |  |  |
| **Motivation** | “Inadequate human resource” | “Inappropriate response to call [after-hours duties] by some clinicians” | “Late coming of staff” | “Late coming [lack of punctuality]” |
|  | “Some clinicians still do not take blood pressure and temperature but write the vital sign names and put a dash” | “Abnormal findings sometimes are not reported in time for interventions to be carried out” | “Absenteeism” | “Staff not recording the observations in patient charts |
|  |  | “Poor attitude of some staff on wards on vitals monitoring” | “Indiscipline among some staff” |  |

**Legend: ***The target behaviors assessed were 1) Collection of vital sign parameters; 2) Diagnosis of severe illness; 3) Targeted management of severe illness conditions.

**Table S3b: Intervention functions targeting identified barriers and facilitators as defined in the Behavioral Change Wheel framework**

| **Intervention** | **Collection of vital sign parameters** | **Diagnosis of severe illness** | **Targeted management of** |
| --- | --- | --- | --- |
| **functions** |  |  | **severe illness conditions** |
| Education | Expert clinicians educate HCW on techniques to collect physiologic vital sign parameters |  | Expert clinicians educate HCW on resuscitative interventions for severe illness conditions |
| Training |  | Expert clinicians simulate diagnosis of severe illness conditions with HCW | Expert clinicians simulate resuscitation of severe illness conditions with HCW |
| Persuasion | Expert clinicians, local champions convince HCW on importance of consistent vital sign collection |  |  |
|  | Audit data on vital sign collection frequency, performance goals provided to HCW |  |  |
| Environmental restructuring | Quality improvement fund provided to purchase equipment for measuring vital signs | Reorganization of health facility to create dedicated triage bay | Reorganization of health facility to create dedicated resuscitation bay |
|  |  | Quality improvement fund provided to repair areas to be used for emergency care |  |
| Enablement | Assign auxiliary staff (cleaners, records, receptionists, mortuary attendants) to collect vital signs during busy periods | HCW re-assigned to cover emergency care areas when duties elsewhere were unoccupied | Enhanced professional identity, belief in capability to change outcomes following successful emergency resuscitation |
| Modeling |  | HCW recount challenges in diagnosing severe illness conditions | Expert clinicians simulate resuscitation of severe illness conditions |
| Incentivization |  |  |  |
| Restriction |  |  |  |
| Coercion | Peer monitoring to ensure vital sign capture |  |  |
|  | Disciplinary action for absenteeism/lateness |  |  |

**Abbreviation:** HCW: health care worker

| **Vital sign** | **%, Pre-intervention**  **period**  **n=1633** | **%, Intervention**  **period**  **n=4126** | **% Difference**  **(95% CI)** | **P-value** |
| --- | --- | --- | --- | --- |
| **Health Facility 1** | **n=759** | **n=1336** |  |  |
| Temperature | 9 | 34 | +25 (+21 to +29) | <0.001 |
| Heart rate | 5 | 33 | +28 (+24 to +31) | <0.001 |
| Blood pressure | 38 | 52 | +14 (+9 to +20) | <0.001 |
| Respiratory rate | 5 | 7 | +2 (+0.2 to +4) | 0.033 |
| Pulse oximetry^*^ | 0 | 23 | -- | -- |
| Mental status | 16 | 21 | +5 (+0.8 to +8) | 0.017 |
| **Health Facility 2** | **n=117** | **n=1018** |  |  |
| Temperature | 90 | 93 | +3 (-15 to +22) | 0.692 |
| Heart rate | 12 | 49 | +37 (+30 to +45) | <0.001 |
| Blood pressure | 93 | 93 | +0.2 (-18 to +19) | 0.987 |
| Respiratory rate | 6 | 14 | +8 (+3 to +13) | 0.002 |
| Pulse oximetry^*^ | 2 | 39 | -- | -- |
| Mental status | 2 | 1 | -0.4 (-3 to +2) | 0.731 |
| **Health Facility 3** | **n=663** | **n=1516** |  |  |
| Temperature | 10 | 32 | +22 (+18 to +26) | <0.001 |
| Heart rate | 10 | 14 | +4 (+0.6 to +7) | 0.018 |
| Blood pressure | 55 | 68 | +13 (+6 to +20) | <0.001 |
| Respiratory rate | 3 | 4 | +1 (-0.2 to +3) | 0.092 |
| Pulse oximetry^*^ | 0.2 | 0.3 | -- | -- |
| Mental status | 11 | 13 | +2 (-0.4 to +6) | 0.898 |
| **Health Facility 4** | **n=94** | **n=256** |  |  |
| Temperature | 50 | 92 | +42 (+24 to +61) | <0.001 |
| Heart rate | 21 | 77 | +56 (+41 to +70) | <0.001 |
| Blood pressure | 61 | 93 | +32 (+13 to +52) | 0.001 |
| Respiratory rate | 10 | 49 | +39 (+29 to +50) | <0.001 |
| Pulse oximetry^*^ | 0 | 55 | -- | -- |
| Mental status | 7 | 33 | +26 (+17 to +35) | <0.001 |

**Table S4a: Between-site variation in vital sign collection before and after SIMS introduction, by site**

**Legend: ^a^**Oximetry was recorded too rarely in the pre-period for the model to be estimated with the site interaction.

**Table S4b: Between-site variation in impact of SIMS on vital sign collection, by vital sign**

| **Vital sign** | **%, Pre-intervention**  **period**  **n=1633** | **%, Intervention**  **period**  **n=4126** | **% Difference**  **(95% CI)** | **P-value** |
| --- | --- | --- | --- | --- |
| **Temperature** |  |  |  | **<0.001^*^** |
| Site 1 | 9 | 34 | +25 (+21 to +29) | <0.001 |
| Site 2 | 90 | 93 | +3 (-15 to +22) | 0.692 |
| Site 3 | 10 | 32 | +22 (+18 to +26) | <0.001 |
| Site 4 | 50 | 92 | +42 (+24 to +61) | <0.001 |
| **Heart rate** |  |  |  | **<0.001^*^** |
| Site 1 | 5 | 33 | +28 (+24 to +31) | <0.001 |
| Site 2 | 12 | 49 | +37 (+30 to +45) | <0.001 |
| Site 3 | 10 | 14 | +4 (+0.6 to +7) | 0.018 |
| Site 4 | 21 | 77 | +56 (+41 to +70) | <0.001 |
| **Blood pressure** |  |  |  | **0.029^*^** |
| Site 1 | 38 | 52 | +14 (+9 to +20) | <0.001 |
| Site 2 | 93 | 93 | +0.2 (-18 to +19) | 0.987 |
| Site 3 | 55 | 68 | +13 (+6 to +20) | <0.001 |
| Site 4 | 61 | 93 | +32 (+13 to +52) | 0.001 |
| **Respiratory rate** |  |  |  | **0.012^*^** |
| Site 1 | 5 | 7 | +2 (+0.2 to +4) | 0.033 |
| Site 2 | 6 | 14 | +8 (+3 to +13) | 0.002 |
| Site 3 | 3 | 4 | +1 (-0.2 to +3) | 0.092 |
| Site 4 | 10 | 49 | +39 (+29 to +50) | <0.001 |
| **Pulse oximetry†** |  |  |  | **--** |
| Site 1 | 0 | 23 | -- | -- |
| Site 2 | 2 | 39 | -- | -- |
| Site 3 | 0.2 | 0.3 | -- | -- |
| Site 4 | 0 | 55 | -- | -- |
| **Mental Status** |  |  |  | **0.016^*^** |
| Site 1 | 16 | 21 | +5 (+0.8 to +8) | 0.017 |
| Site 2 | 2 | 1 | -0.4 (-3 to +2) | 0.731 |
| Site 3 | 11 | 13 | +2 (-0.4 to +6) | 0.898 |
| Site 4 | 7 | 33 | +26 (+17 to +35) | <0.001 |

**Legend: ^*^**Bolded p-values represent test of site-time interaction; **^†^**Oximetry was recorded too rarely in the pre-period for the model to be estimated with the site interaction.

**Additional file 1 - Figure Legends**

Figure S1: Diagram illustrating the conceptual model based on components of the COM-B model that was utilized to develop the SIMS intervention.

Figure S2: Staggered, pre-post quasi-experimental study design utilized for implementation of SIMS intervention. Baseline period indicates time period following Quick Check+ training and before SIMS intervention. Intervention period indicates time period during which SIMS intervention was implemented.

Figure S3: Flow diagram for patients included in study.

**Additional file 1 – Figures**

**Figure S1**

**
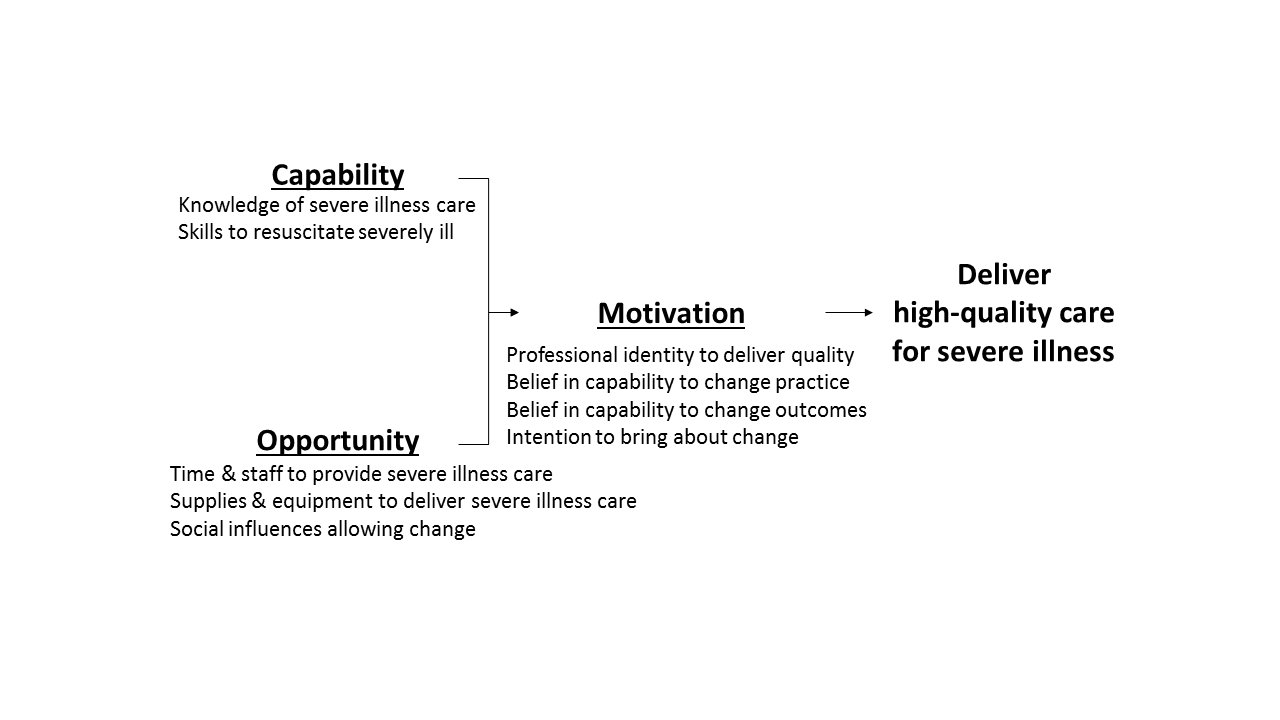
**

**Figure S2**

**
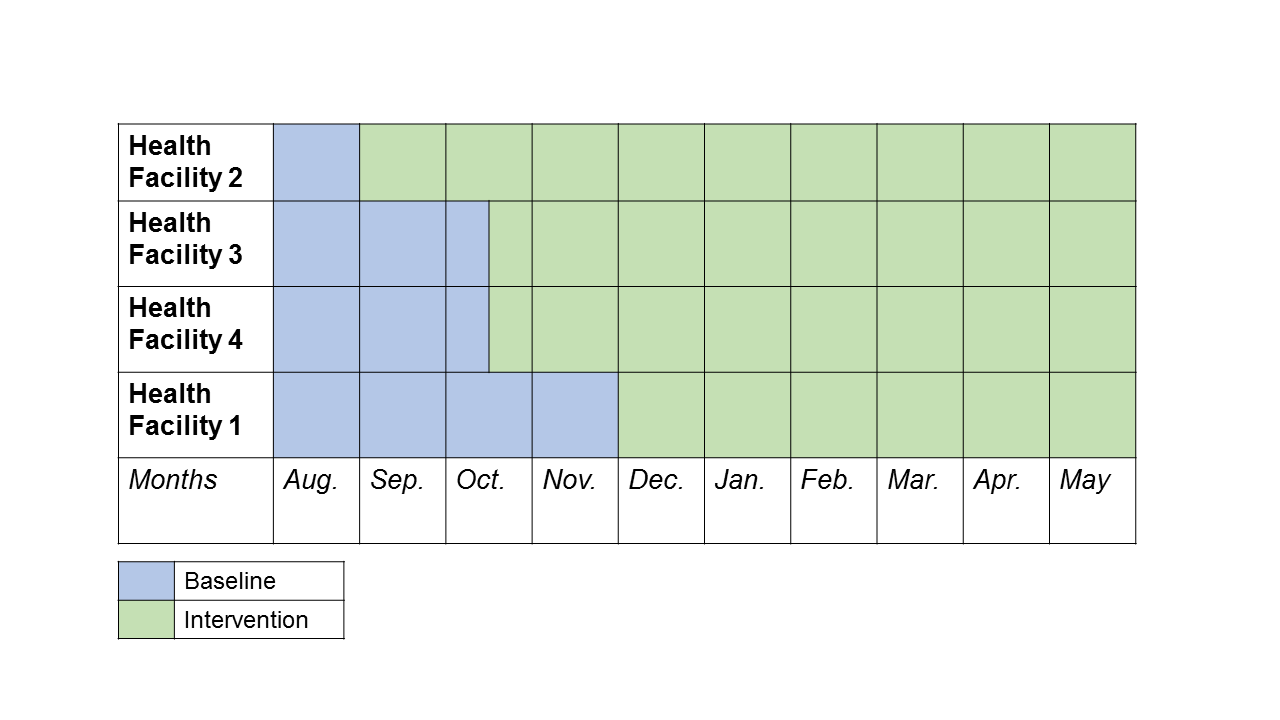
**

**Figure S3**

**
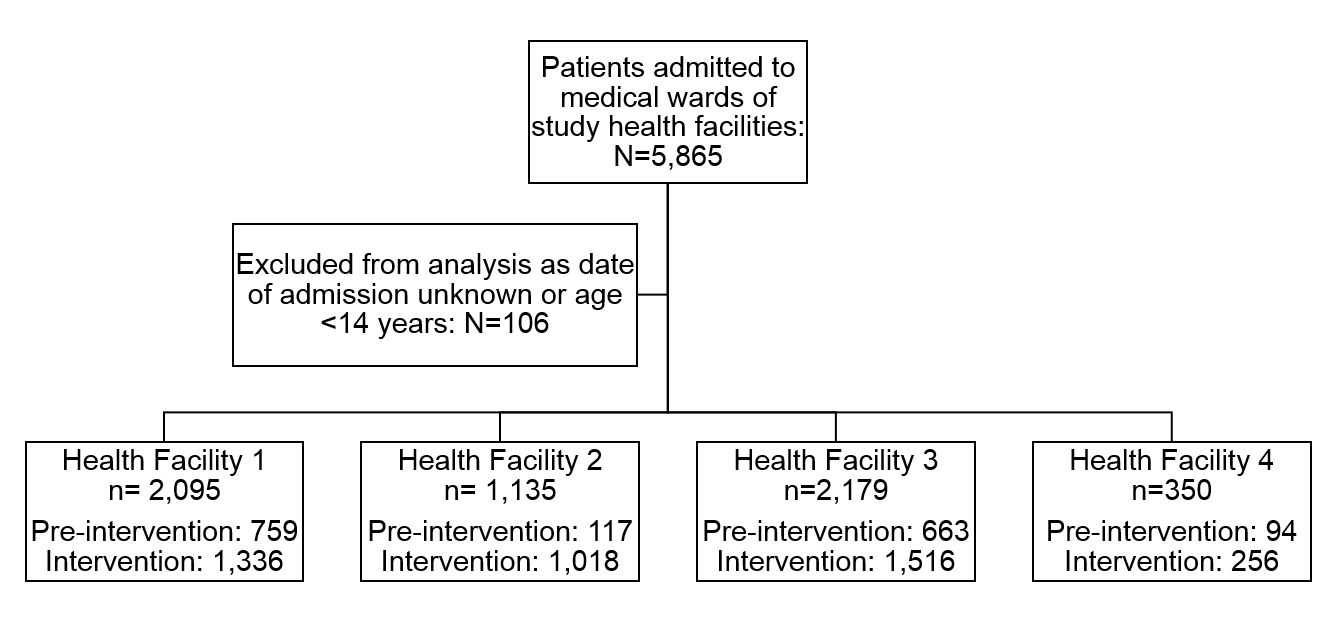
**
